# HSP90AA1 promotes lymphatic metastasis of hypopharyngeal squamous cell carcinoma by regulating epithelial-mesenchymal transition

**DOI:** 10.32604/or.2023.030081

**Published:** 2023-07-21

**Authors:** FENGXIANG TANG, YANSHI LI, MIN PAN, ZHIHAI WANG, TAO LU, CHUAN LIU, XIN ZHOU, GUOHUA HU

**Affiliations:** Department of Otolaryngology, The First Affiliated Hospital of Chongqing Medical University, Chongqing, China

**Keywords:** Hypopharyngeal squamous cell carcinoma, Lymphatic metastasis, HSP90AA1, EMT, HNSCC

## Abstract

**Background:**

Lymphatic metastasis (LM) emerges as an independent prognostic marker for hypopharyngeal squamous cell carcinoma (HSPSCC), chiefly contributing to treatment inefficacy. This study aimed to scrutinize the prognostic relevance of HSP90AA1 and its potential regulatory mechanism of concerning LM in HPSCC.

**Methods:**

In a preceding investigation, HSP90AA1, a differential gene, was discovered through transcriptome sequencing of HPSCC tissues, considering both the presence and absence of LM. Validation of HSP90AA1 expression was accomplished via qRT-PCR, western-blotting(WB), and immunohistochemistry(IHC), while its prognostic significance was assessed employing Kaplan–Meier survival analysis(KMSA), log-rank test(LR), and Cox’s regression analysis(CRA). Bioinformatics techniques facilitated the prediction and analysis of its plausible mechanisms in LM, further substantiated by *in vitro* and *in vivo* experiments utilizing FaDu cell lines.

**Results:**

HSP90AA1 is substantially up-regulated in HPSCC with LM and is identified as an independent prognostic risk determinant. The down-regulation of HSP90AA1 can achieve inhibition of tumor cell proliferation, migration and invasion. Both *in vivo* experiments and Bioinformatics exploration hint at promoting LM by Epithelial-mesenchymal transition (EMT), regulated by HSP90AA1.

**Conclusions:**

HSP90AA1, by controlling EMT, can foster LM in HPSCC.This finding sets the foundation for delving into new therapeutic targets for HPSCC.

## Introduction

Among head and neck malignancies, HPSCC ranks as one of the most severe constituting approximately 0.8%–1.5% of these carcinomas [1]. Recently, laryngeal and nasopharyngeal cancer incidence among malignant head and neck tumors has decreased gradually, while the incidence of HPSCC shows an annual increase and has a trend in younger. Due to its unique anatomical features, HPSCC is characterized by concealed onset and great difficulties in diagnosis. Regrettably, most patients with HPSCC are identified in the advanced stages of the disease, and a significant proportion of them, ranging from 60% to 80%, exhibit lymph node metastasis (LNMs) at the point of initial diagnosis [2]. Despite advances in diagnosing and treating head and neck malignant tumors, including standardized surgical methods, radiotherapy and chemotherapy, and gradually maturing targeted therapy and immunotherapy [3,4], the HPSCC prognosis remains poor. Specifically, the five-year survival rate (5YSR) of HPSCC patients receiving radical surgery accompanied by radical radiotherapy was 25%–40%, which is lower than the average 5YSR (50%) of malignant tumors of head and neck, while the 5YSR of HPSCC patients receiving radiotherapy or chemotherapy only was 12%–14% [5–9].

It has been demonstrated that LNMs was a factor influencing HPSCC prognosis, as well as the reason behind the failed treatment of HPSCC [9–11]. Meanwhile, the size and quantity of metastatic lymph nodes (MLNs), and extracapsular extension can impact the HPSCC patients’ prognosis [11–14]. Multivariate analysis of factors influencing HPSCC prognosis revealed that LNMs isare an independent HPSCC prognostic risk factor [15]. Therefore, the exploration of molecular markers and mechanisms driving LNMs in HPSCC holds substantial theoretical and clinical relevance. This effort can aid in advancing personalized treatment strategies for HPSCC patients, enhancing their survival rates (SRs). It also serves as an invaluable guide for clinicians in treatment decisions for HPSCC patients, including the extent of lymph node resection and the selection of tumor-specific therapeutic agents.

Tumor LNMs isare a complex pathological process. Tumor cells can migrate by reducing cell adhesion, increasing cell mobility, and destroying the extra matrix cellular. As cancer advances, epithelial tumor cells can undergo a profound transformation referred to as EMT. This process induces significant alterations in the characteristics of neoplastic cells, resulting in the disappearance of epithelial markers such as E-cadherin, modifications, in cell polarity and intercellular connections, and an elevation in mesenchymal markers, such as N-cadherin, fibronectin, and vimentin. This process enhances the ability of cancer cells to undergo EMT, leading to increased detachment from the primary tumor and invasiveness, which are essential for the initiation of metastasis. As a result, neoplastic cells have the ability to invade the circulatory or lymphatic system and from metastases in remote locations. This first step in metastasis involves epithelial cells losing their polarity and intercellular connections and acquiring enhanced migration and invasion capacities [16–18]. Transcription factors like SNAIL, SLUG, TWIST, and ZEB families trigger the transcriptional reprogramming that drives EMT. This leads to subsequent E-cadherin suppression and up-regulation of VIMENTIN and N-cadherin, reducing epithelial cell-cell adhesion and promoting cancer cell invasion and migration [19,20]. Contrarily, impeding EMT results in the E-cadherin up-regulation and down-regulation of VIMENTIN, N-cadherin, SLUG, and SNAIL. Various studies suggest that EMT can be regulated at the post-transcriptional level, either promoting or inhibiting mesenchymal cell characterization and tumor cell metastasis [21–23]. However, the specific molecular mechanism of EMT in HPSCC and the role of HSP90AA1 in regulating EMT in cancer cells are areas that warrant further exploration.

Transcriptome sequencing, an efficient, mature, and high-throughput technique primarily used to explore gene functions, develop new targeted therapies, and validate tumor biomarkers, has been employed in past studies [24]. This technique revealed through sequencing of clinical samples that HSP90AA1 is an independent prognostic marker for LNMs in HPSCC [25,26].

The HSP90AA1 gene encodes heat shock protein α (HSP90α), and its content is rich, accounting for 1%–2% of cells [27,28]. HSP90α exists in an inactive form in normal cells, but in an active form in tumor cells [29,30]. The activated HSP90α can stabilize downstream proteins, such as mutant P53, EGFR, BRAF, AKT, MET, VEGFR, FLT3, HIF-1αn, HER-2, and hTERT [31], and then indirectly accelerate the progress of tumors. It has been demonstrated that the increased HSP90α protein was significantly associated with lymphoid infiltration [32]. Research on colorectal cancer, ovarian cancer, and renal cancer showed that HSP90α could regulate EMT pathway, and downregulate its epithelial markers E-cadherin and Vimentin [33,34], resulting in tumor LNMs. Unfortunately, the precise mechanism of HSP90AA1 in HPSCC and EMT has not been fully investigated.

To summarize, LM significantly impacts the prognosis of HPSCC patients. The current research aims to elucidate the role and regulatory mechanism of HSP90AA1 in influencing LM development in HPSCC. We aim to lay a foundation for discovering innovative therapeutic targets by accomplishing this. In the long run, this endeavor is anticipated to provide fresh insights for establishing intelligent diagnostic approaches and precision medicine for treating HPSCC.

## Methods

### Expression and prognostic value

#### Patients and tissue samples

In this study, HPSCC and lymph node tissues were collected from 59 cases diagnosed with HPSCC and received surgical resection by the Department of Otolaryngology of the First Affiliated Hospital of Chongqing Medical University (FAH-CQMU) from 2012 to 2017. Inclusion criteria: cases were diagnosed pathologically as HPSCC; Tumor node metastasis (TNM) classification was staged according to the American Joint Committee on Cancer (AJCC) 8^th^ edition; the first diagnosis was hypopharyngeal carcinoma, without other malignant tumor complications; patients received no preoperative targeted therapy, chemotherapy, or radiotherapy; patients had complete follow-up records. The procedure for gathering data adhered to the guidelines outlined in the Declaration of Helsinki, with each participant providing informed consent, and was approved by the Ethics Committee of the FAH-CQMU.

Few fresh samples were promptly preserved in liquid nitrogen, while others were initially maintained in formalin before being embedded in paraffin. Two experienced pathologists identified the tissue composition, pathological stage, differentiation grade, status of extracapsular extension and LNMs. Table 1 summarizes clinical features of the patients.

**Table 1:** The clinicopathological features of patients with hypopharyngeal squamous cell carcinoma

Features	Factors	No. (%)
Age	<60	23 (38.98%)
	≥60	36 (61.02%)
Gender	Male	58 (98.31%)
	Female	1 (1.69%)
Stages	I	0
	II	3 (5.08%)
	III	19 (32.21%)
	IV	37 (62.71%)
T stage	T1	3 (5.08%)
	T2	9 (15.25%)
	T3	28 (47.47%)
	T4	19 (32.20%)
Lymphatic metastasis	Presence	39 (66.10%)
	Absence	20 (33.90%)
N stage	N0	19 (32.20%)
	N1	8 (13.56%)
	N2	26 (44.07%)
	N3	6 (10.17%)
Pathological differentiation	Low	14 (23.73%)
	Moderate and high	45 (76.27%)
Extracapsular extension	Yes	10 (16.95%)
	No	49 (83.05%)
Drinking	Yes	42 (71.19%)
	No	17 (28.81%)
Smoking	Yes	49 (16.95%)
	No	10 (16.95%)

#### Gene sequencing and qRT-PCR

In our previous experiments, RNA was extracted from primary tissues of 10 HPSCC cases (five HPSCC cases with LNMs and five HPSCC cases without LNMs), followed by gene sequencing in Jingzhou Gene Technology Co., Ltd. (China) to identify the differential gene HSP90AA1. Following the guidelines provided by the manufacturer, total RNA was isolated from HPSCC primary tissues employing an RNA Extraction Kit (Takara, Dalian, China). This was followed by converting RNA into cDNA, a procedure facilitated by using a Reverse Transcription Kit (Takara). An SYBR Primer RT-PCR Kit (Takara) was employed for the next step. GAPDH served as the internal control, with relative quantification of expression calculated using the 2^−ΔΔCt^ method. The primer sequences used in this procedure are detailed in Table 2.

**Table 2:** Primer sequences

NAME	Primer sequences (5′~3′)
HSP90AA1-F	TATAAGGCAGGCGCGGGGGT
HSP90AA1-R	TGCACCAGCCTGCAAAGCTTCC
E-cadherin-F	TGC CCA GAA AAT GAA AAA GG
E-cadherin-R	GTG TAT GTG GCA ATG CGT TC
N-cadherin-F	GAC AAT GCC CCT CAA GTG TT
N-cadherin-R	GAC AAT GCC CCT CAA GTG TT
Vimentin-F	GAG AAC TTT GCC GTT GAA GC
Vimentin-R	GCT TCC TGT AGG TGG CAA TC
Snail-F	GCG AGC TGC AGG ACT CTA AT
Snail-R	CCT CAT CTG ACA GGG AGG TC
Slug-F	TGA TGA AGA GGA AAG ACT ACAG
Slug-R	GCTCACATATTCCTTGTCACAG
GAPDH-F	GAAGGTGAAGGTCGGAGTC
GAPDH-R	GAAGATGGTGATGGGATTTC

Abbreviations: F, forward; R, reverse.

#### Protein separation and WB

Proteins were harvested from HPSCC tumor tissues and corresponding normal tissues (comprising seven samples each from patients with LNMs, seven samples from patients without lymph node metastasis, and 7 samples from corresponding normal tissues) utilizing the Total Protein Extraction Kit (KeyGen Biotech, China). These protein lysates were subjected to a 15% sodium dodecyl sulfate-polyacrylamide gel, and transferred onto polyvinylidene fluoride (PVDF) membranes. These membranes underwent nocturnal incubation at 4°C in the diluted primary antibody solution. The anti-HSP90AA1 and anti-GAPDH primary antibodies (Abcam, UK) were diluted at 1:1000 and 1:300. GAPDH was used as the reference, and then PVDF membranes were immersed in goat anti-rabbit LgG (Beyotime, Shanghai, China, 1:5000) diluent, followed by washing thrice in PBST, 10 min each time on a shaker. Protein band signals were detected by High-Efficiency Chemiluminescence ECL Kit (Termo, Shanghai, China), and ChemiDoc Touch Imaging System (BIO-RAD, Hercules, CA, USA) was used for visualization.

#### Immunohistochemistry

The IHC staining was performed on as-prepared paraffin sections (4 μm), including 59 primary tumor tissues from HPSCC patients (21 HPSCC cases without LM, 38 HPSCC cases with LM). After dewaxing in xylene and hydrating in gradient ethanol, the antigens were recovered in 100°C citrate buffer for 30 min. Then, the sections were blocked with peroxidase for 15 min and washed with PBS 3 times. Anti-HSP90AA1 primary antibody was diluted at 1:200 and incubated in a 4°C refrigerator on a shaker overnight. Following a wash with PBS three times, the sections were subjected to a 5-min staining with Diaminobenzidine reagent followed by a counterstaining step with hematoxylin for approximately 30 s. Furthermore, the specimens underwent dehydration using xylene and a gradient of alcohol and were subsequently covered with coverslips. Tissues treated by PBS, rather than the primary antibody, were used as the negative control (NC). The IHC outcomes were assessed by two proficient pathologists individually. The evaluation was based on the staining intensity, area, and proportion of tumor cells exhibiting a statistically significant positive reaction. The expression of HSP90AA1 was categorized as either negative or positive based on the intensity of staining observed. Tumor cells exhibiting a staining intensity of brown or dark brown and comprising more than 50% of the sample were classified as positive, while those with a staining intensity of yellow or light yellow and comprising 50% or less of the sample were classified as negative. The mean Integral optical density (IOD) was utilized to ascertain the density of positive staining. The average IODs in every image were quantified and tallied using Image-Pro Plus v6.0 software (Media Cybernetics, Inc., USA).

#### Clinical prognostic value analysis

According to the clinical features of HPSCC patients (Table 1) and IHC results, KMSA, LR, and CRA were performed to explore the correlation between HSP90AA1 expression and other clinicopathological characteristics, and between clinicopathological features of HPSCC and the patient’s prognosis. Survival curves were generated to examine the impact of clinicopathological characteristics on the overall survival (OS) of HPSCC patients. Ultimately, an assessment was conducted to determine the independent risk factors that impact the prognosis of patients with HPSCC.

### Bioinformatics analysis

Transcriptome RNA sequencing data, in conjunction with clinical data for 419 cases of head and neck squamous cell carcinoma (HNSCC), were obtained from The Cancer Genome Atlas (TCGA) database (https://cancergenome.nih.gov/). The differential expression of the HSP90AA1 gene in HNSCC was ascertained between normal samples and those with or without LM, utilizing the limma software package in R (http://Bioconductor.org/packages/limma/). Concurrently, differentially expressed genes (DEGs) were assessed within two groups distinguished by high and low expression HSP90AA1 expression. Using STRING (http://www.string-db.org/), the protein-protein interaction (PPI) was analyzed between HSP90AA1 and the aforementioned genes. The resultant PPI network was visually scrutinized using Cytoscape software, and core genes were identified employing the CytoHubba plugin whin the Cytoscape software. To gain insight into the functions and pathways enriched by HSP90AA1 that may facilitate metastasis, a Gene Ontology (GO) enrichment analysis was performed on these core genes.

### Vitro cell experiments

#### Cell culture

The FaDu cell line was procured from the Center for Molecular and Cellular Sciences, Chinese Academy of Sciences (Shanghai). These cells underwent cultivation in a humidified incubator at 37°C in an environment enriched with 5% CO_2_. The medium employed was Dulbecco’s Modified Eagle Medium (DMEM, Gibco, Carlsbad, CA, USA), supplemented with 15% fetal bovine serum (FBS) (Gibco, Carlsbad, CA, USA).

#### Lentivirus transfection

The FaDu cells underwent transfection with lentivirus sh-RNA, targeting HSPA90AA1 for the knockdown, with the lentivirus acquired from GeneChem (Shanghai, China). The lentivirus vector employed was hU6-MCS-Ubiqutin-EGFP-IRES-puromycin. Cells subjected to transfection were divided into sh-NC (knockout negative control) and sh-HSP90AA1 (the knockdown groups). The FaDu cells were grown in 6-well plates, and when they occupied 50%–60% of the plate’s bottom surface, they were exposed to the lentivirus (Multiplicity of Infection, MOI = 10) in accordance with the instruction manual. Post transfection (24 h later), the medium was switched out for DMEM cell culture medium, enriched with 15% FBS. After an additional 48 h of uninterrupted cultivation, puromycin (2 μg/mL) was introduced to stabilize the cells that had been infected. Eventually, the cells were gathered for WB and qRT-PCR (using the method described previously) to assess HSP90AA1 expression in the two groups of FaDu cells.

#### WB and qRT-PCR

The sh-nc-FaDu cells (Lentivirus transfection reagent negative control Fadu cell line) and sh-HSP90AA1-FaDu cells (Lentivirus transfected FaDu cell line with HSP90AA1 knockdown) were collected for WB and qRT-PCR (the same method as above) to detect the expression of HSP90AA1 (Abcam, UK,1:1000), E-cadherin (Abcam, UK,1:1000), N-cadherin (Abcam, UK,1:1000), snail (Abcam, UK,1:1000), slug (Abcam, UK,1:1000) and VIMENTIN (Abcam, UK,1:1000). The primer sequence is shown in Table 2. The anti-GAPDH primary antibodies (Abcam, UK) were diluted at 1:300.

#### EDU diffusion

The stably transfected sh-nc-FaDu cells and sh-HSP90AA1-FaDu cells were inoculated into 12-well plates, respectively. When the adherent area of cells exceeded 80% of the bottom of the plate, the cell proliferation rate was measured by EDU Test Kit (RiBoBio, Guangzhou, China). After staining, images were obtained through the inverted fluorescence microscope.

#### Flow cytometry

For the apoptosis experiment, stably transfected sh-nc-FaDu cells and sh-HSP90AA1-FaDu cells were cultured in 25 cm cell culture flasks, respectively. After the adherent area reached 90% of the bottom of the flask, all cells were collected, respectively. Post-incubation with a binding buffer inclusive of V-FITC/PI, cell samples underwent analysis *via* flow cytometry (Biosciences, CA, USA). The cell cycle was determined by trypsinization. After the two groups of cells were collected, 70% cold ethanol was added for fixation, and the cells were kept overnight in a 4°C refrigerator. Subsequent to the cells’ exposure to RNase A and propidium iodide, flow cytometry was employed the next day to ascertain the cell cycle.

#### Transwell migration and invasion assays

The 24-well plate Transwell chamber with 8 μm pore membrane (Corning, CA, USA) was used for the cell migration experiment. The suspensions of sh-nc-FaDdu cells and sh-HSP90AA1-FaDu cells were prepared and into the Transwell’s upper chamber, followed by the DMEM medium containing 15% FBS added to the lower chamber. Following a standard 24-h culture period, the Transwell chamber was removed, and the culture medium was discarded. This was succeeded by two cycles of rinsing with PBS. Cells adhering to the lower side of the polycarbonate membrane were then fixed with 4% polymethyl alcohol for 30 min at ambient temperature, after which they were stained with 0.5% crystal violet in methanol for 15 min, also at room temperature. Any residual cells present in the upper chamber of the polycarbonate membrane were gently eliminated using a cotton swab. After PBS washing 3 times, images were taken under the inverted fluorescence microscope, and three visual fields in each hole were randomly selected for counting. Likewise, the 24-well plate Transwell chamber with 8-μm pore membrane (Corning) was used for the cell invasion experiment. Overnight, the Matrigel (Biosciences, MA, USA) was allowed to thaw at a temperature of 4°C. It was diluted to a 1 mg/mL concentration using a pre-chilled, serum-free DMEM medium. This diluted Matrigel was carefully deposited onto the central region of the upper chamber’s base within the Transwell chamber. It was then placed in a 37°C incubator for a duration of 5 h to allow for gel formation. Cell seeding was performed, and cells adhering to the lower side of the polycarbonate membrane were subjected to fixation and staining, employing the previously detailed method. To conclude, using an inverted fluorescence microscope, three random visual fields from each well were selected for counting.

#### Wounding healing assay

Parallel horizontal lines were drawn on the back of 6-well plates, with each hole passed through 3 horizontal lines. Then, sh-HSP90AA1-FaDu cells and sh-nc-FaDu cells were uniformly inoculated into the plates and were cultured in DMEM cell culture medium containing 15% FBS overnight until the cell adherent area roughly reached 100% of the bottom of each well. The pipette tip was used to draw lines at the bottom of the petri dish perpendicular to the black horizontal line. Then, exfoliated cells were cleansed with PBS to enhance the visibility of the scratches. This was followed by incubation in a serum-free culture medium maintained at 37°C. After specific intervals of 0, 24, and 48 h, cell samples were retrieved, followed by observation and image capture utilizing an inverted fluorescence microscope. Three cell scratches in each well were selected randomly to calculate the wound healing rate using the ImageJ software.

### Establishment of xenotransplantation model of nude mouse foot

Male nude mice, specifically four-week-old BALB/cA-nu were procured from Tengxin Biological Science Co., Ltd. based in Beijing, China. In each group of five mice, the left foot pad was injected with either stably transfected sh-nc-FaDu cells or sh-HSP90AA1-FaDu cells, with the cell count approximating 1 × 106. Subsequent to the injection, the nude mice were kept in a specific pathogen-free (SPF) environment within the CQMU animal center for 28 days. Upon visible tumorigenesis at the foot pad, the mice were humanely euthanized. Tumors and MLNs from the foot pad were collected, and the tumor volume and mass were quantified. The tumor volume was computed using the formula: tumor volume = [length × (width)^2^] × π/6 [30]. Portions of the tissues were set aside for protein and total RNA extraction, while the remainder was fixed in 4% paraformaldehyde. Techniques like qRT-PCR, WB, and IHC, previously elaborated, were deployed to determine HSP90AA1 expression levels. The Ethics Committee of the FAH-CQMU sanctioned all mouse experiments, and they were executed in alignment with the United Kingdom’s Animals (Scientific Procedures) Act and the guidelines stipulated by the National Institutes of Health of the United States.

### Statistical analysis

All IHC, WB, qRT-PCR, EDU, Transwell tests, flow cytometry, and animal tests were repeated more than 3 times, and more than three pictures or visual fields in the same group were randomly selected for data analysis. SPSS 22.0 and GraphPad Prism 6.0 were used for statistical analysis. The association between HSP90AA1 and E-Cadherin expression in IHC and the clinicopathological characteristics of HPSCC was assessed through the Chi-square test. The protein expression level in WB was analyzed with ImageJ, and qRT-PCR results of three groups were analyzed with one-way ANOVA. KMSA, LR, and CRA were used to evaluate the prognostic factors. All data were expressed as mean ± SD, and *p* < 0.05 was considered statistically significant.

## Result

### Expression of HSP90AA1 in HPSCC tissues

#### The mRNA expression of HSP90AA1 in LM patients with HPSCC was significantly up-regulated

**Figure 1:**
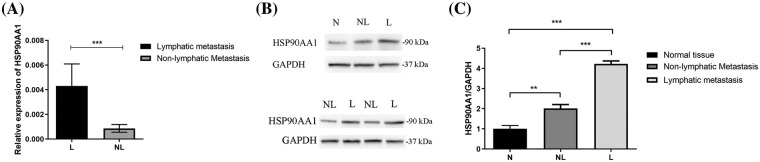
RT-PCR and Western blot results for tissues. (A) The mRNA expression level of HSP90AA1 in RT-PCR (11 samples from HPSCC patients with LM and another 11 from HPSCC patients without LM). (B) The expression of HSP90AA1 protein level by Western blot assay (7 samples from patients with LM, 7 samples from patients without LM, and 7 samples from adjacent normal tissues). (C) Protein expression levels were analyzed by ImageJ software. *GAPDH* served as an internal reference. The vertical axis represents the relative protein expression level of HSP9AA1, and the horizontal axis represents the different groups. Abbreviations: N: Normal tissues; NL: tumor tissues from non-lymphatic metastasis patients; L: tumor tissues from lymphatic metastasis patients. **p < 0.01, ***p < 0.001. All the data are presented as mean ± SD from three independently performed experiments.

The study employed 11 sets of tumor tissues, each comprising one sample from a patient with LM and another from non-LM patients for RT-PCR analysis. The findings, displayed in Fig. 1A, indicated a significant elevation in the relative mRNA expression of HSP90AA1 in the LM group compared to the non-LM group, with the variance being statistically significant (*p* < 0.001).

#### Protein expression of HSP90AA1 escalates in HPSCC patients with LM

Fig. 1B illustrates protein band signals, representing the level of protein expression. Consistent with the RT-PCR results, the expression of HSP90AA1 in tumor tissues of LM patients markedly surpassed that in the non-LM group. Using ImageJ for the evaluation of protein expression levels (Fig. 1C), a statistically significant difference was observed (*p* < 0.01, *p* < 0.001).

#### Determining HSP90AA1 expression through IHC

**Figure 2:**
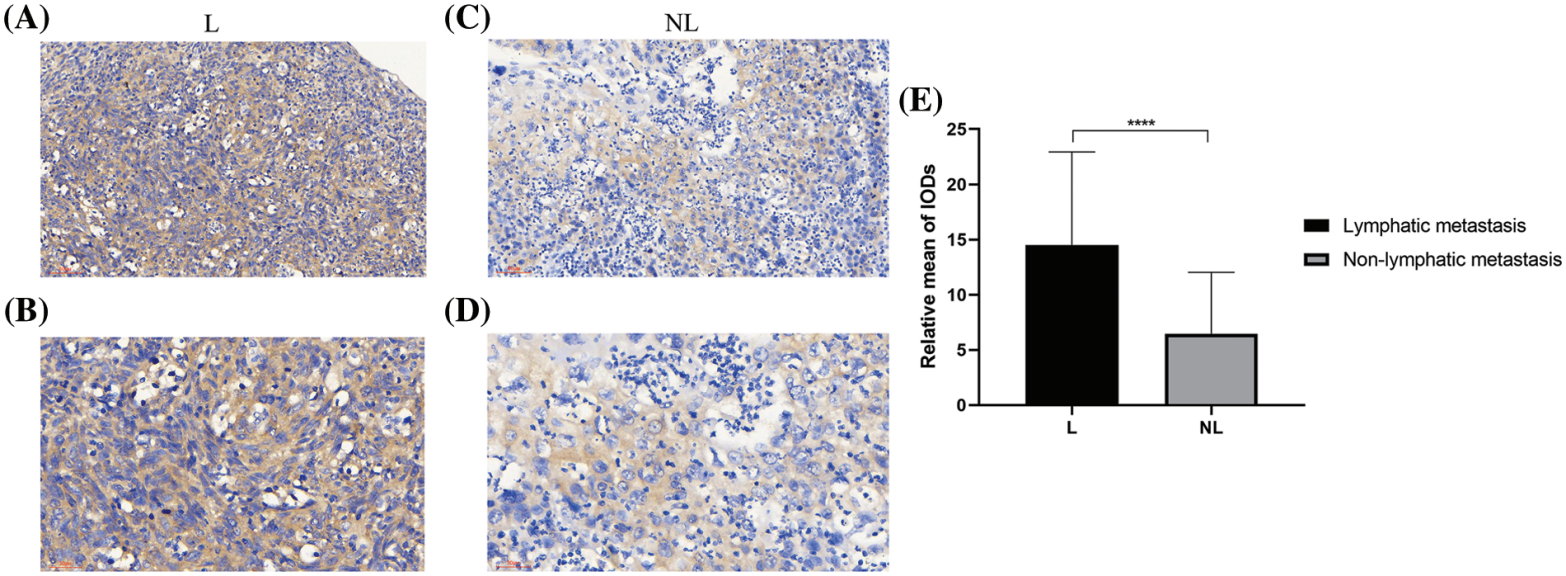
The immunohistochemical results of HSP90AA1 protein in HPSCC tissues were as follows: Expression levels of HSP90AA1 protein in HPSCC tissues from patients with LM ((A) ×200 and (B) ×400). HPSCC tissues from patients without LM ((C) ×200 and (D) ×400). (E) The mean of IOD of HPSCC tissues from patients with or without LM. Abbreviations: NL: tumor tissues from non-LM patients; L: tumor tissues from lymphatic metastasis patients; IOD: Integral optical density. ****p < 0.0001. All the data are presented in the form of mean ± SD from three independently performed experiments.

IHC analysis of HSP90AA1 protein expression was conducted on HPSCC samples sourced from 21 patients without LM and 38 patients with LM. The data revealed higher expression of HSP90AA1 in tumor tissues of LM patients (Figs. 2A and 2B) as opposed to non-LM patients (Figs. 2C and 2D). The LM group also displayed a significantly higher rate of positive HSP90AA1 (30/38, 78.9%) compared to the non-LM group (7/21, 33.3%) (Table 3). The mean of IODs was measured and counted using Image-Pro Plus v6.0 software, indicating a statistically significant difference (Fig. 2E).

**Table 3:** The immunohistochemical expression of HSP90AA1 in clinical specimens of patients

Tissue	Number of patients	Expression of HSP90AA1		*p* value*
		Positive	Negative	
Primary tumor in patients with lymphatic metastasis	38	30	8	*p* < 0.001
Primary tumor in patients without lymphatic metastasis	21	7	14	

Note: *p values are from χ2 test or Fisher’s exact test and were statistically significant when <0.05.

### Clinical prognostic value analysis

#### The impact of LM on HPSCC patient survival

**Figure 3:**
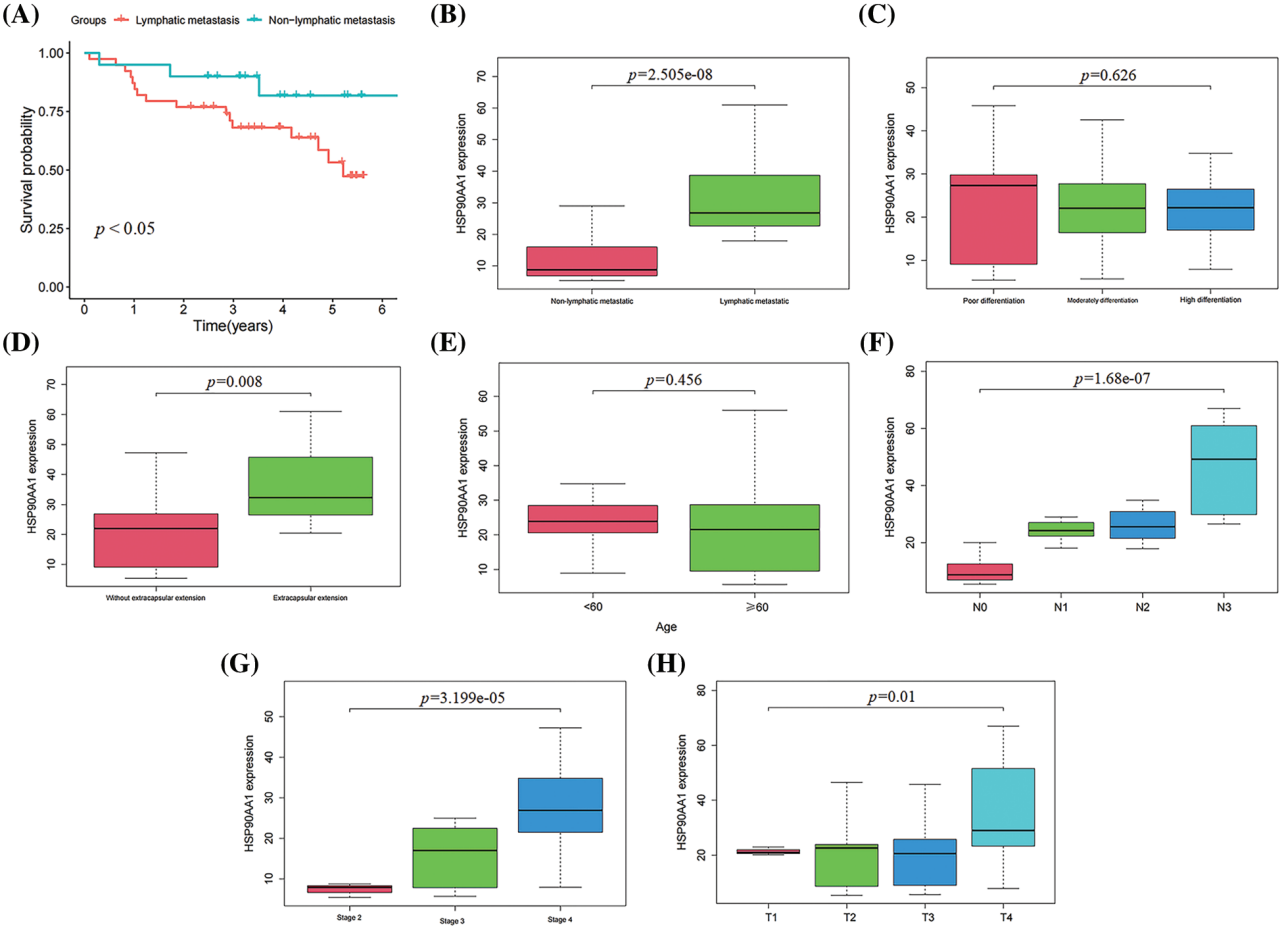
Association of HSP90AA1 expression with clinical features. (A) Correlations between LM and overall survival in patients with HPSCC. (B) lymphatic metastases; (C) pathological differentiation degree; (D) extracapsular extension; (E) age; (F) N stage; (G) clinical stage; (H) T stage.

LM has been established as a key determinant of the survival rate (SR) for HPSCC patients. When compared with HPSCC, patients without LM, those with LM manifested significant reductions in SRs (a decrease of 15% in 3-year SR and 25% in 5-year SR) (*p* < 0.05, Fig. 3A). The median duration of follow-up for all patients was 3.56 years.

#### Associations between HSP90AA1 expression and clinical features

By analyzing the clinical characteristics of 59 patients with HPSCC (from the FAH-CQMU from 2012 to 2017), including age, sex, clinical stage, LNMs, TMN stage, extracapsular extension, pathological differentiation degree, HSP90AA1 expression (mean of IOD), Survival state and survival time. As shown in Figs. 3B–3H, the expression of HSP90AA1 in HPSCC tissues of metastatic and non-metastatic patients was significantly different, and the expression of HSP90AA1 was increased in the metastatic group (*p* < 0.0001). HSP90AA1 expression was also associated with clinical staging (*p* < 0.0001), T stage (*p* < 0.0001), N stage (*p* < 0.0001), and whether there is an episcopal infiltration (*p* < 0.0001) significantly associated.

#### Prognostic value of HSP90AA1 expression in HPSCC

**Figure 4:**
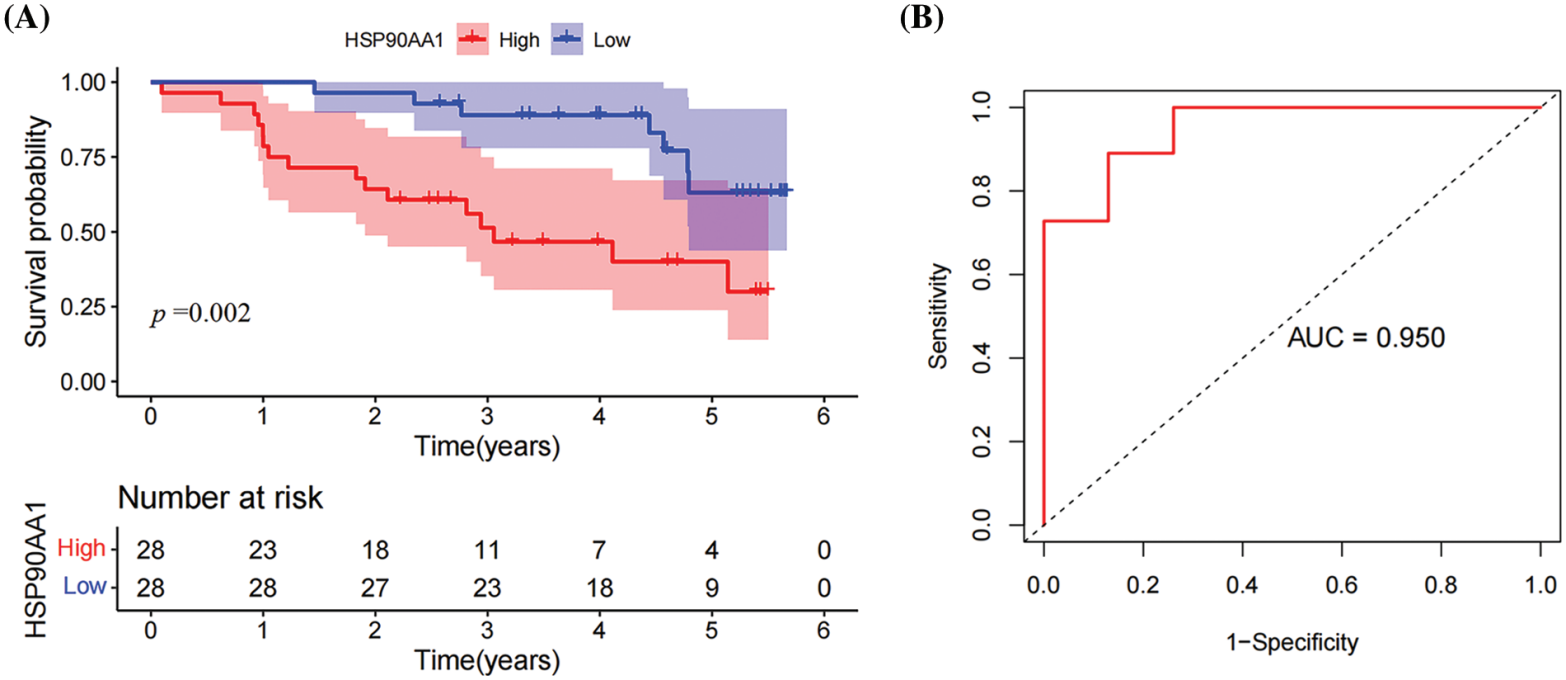
Prognostic value of HSP90AA1 in HPSCC. (A) Correlations between HSP90AA1 expression and overall survival in patients with HPSCC. The horizontal axis represents survival time (years), and the vertical axis represents the percent survival. The upper line shows the survival rate of patients with low expression of HSP90AA1, while the bottom line represents patients with high expression of HSP90AA1. (B) Receiver operating characteristic (ROC) curve for HSP90AA1 expression in HPSCC.

Through the application of KMSA, LR, and CRA, it was discovered that the HSP90AA1 expression serves as an independent prognostic risk factor for HPSCC patients in comparison to other clinicopathological characteristics (HR *>* 1, *p* < 0.05) (Table 4 and Fig. 4A). The median duration of patient follow-up was 42.7 months. In order to ascertain the prognostic significance of HSP90AA1, a Receiver Operating Characteristic (ROC) curve was generated using the expression data from a patient pool comprising 21 individuals without LM and 40 with LM. The area under the ROC curve was determined to be 0.950, signifying a substantial diagnostic potential (Fig. 4B).

**Table 4:** Univariate and multivariate Cox regression analyses of overall survival in hypopharyngeal squamous cell carcinoma patients

Variable	Univariate analyses			Multivariate analyses		
	HR	95% CI	*p* ^#^	HR	95% CI	*p* value^#^
Age	1.002	0.954–1.053	0.931			
Drinking	1.522	0.505–4.592	0.456			
T stage	2.560	1.154–5.678	0.021			
Lymphatic metastasis	**1.968**	**0.744–5.206**	**0.024**	**1.591**	**1.322–1.914**	**0.029**
Pathological differentiation	0.727	0.406–1.304	0.284			
Extracapsular extension	2.038	0.744–5.583	0.166			
Clinical stage	2.584	0.892–7.485	0.008			
Expression of HSP90AA1	**1.148**	**1.092–1.205**	**<0.001**	**1.238**	**1.119–1.369**	**<0.001**

Note: ^#^*p* values from Cox analyses were statistically significant when <0.05.

### HSP90AA1 promotes LM by regulating EMT

**Figure 5:**
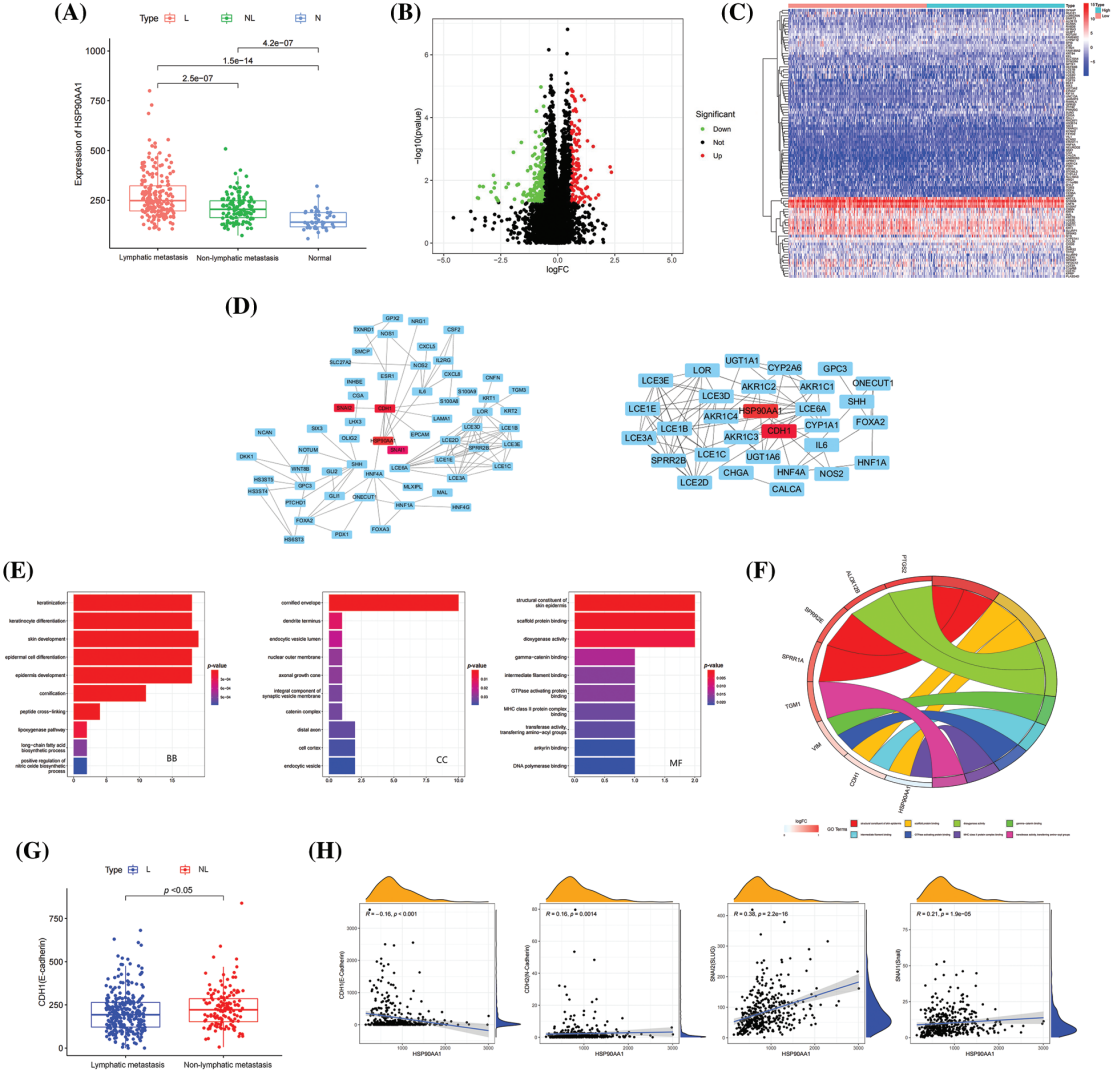
Differential pathway analysis by bioinformatics. (A) The expression level of HSP90AA1 in HNSCC with or without LM and normal samples. (B) Volcano map of DEGs, the fold change (FC) threshold was set as |log2FC| > 0.5, *p* < 0.05. (C) Heat map of DEGs. (D) PPI network of genes involved in the cancer biological processes with both HSP90AA1 and CDH1. (E) GO terms analysis. (F) Circos diagram of MF. (G) The expression level of CDH1 in HNSCC with or without LM. (H) The association between HSP90AA1 and CDH1, CDH2, SNAI1, and SNAI2 expression. Abbreviations: GO, Gene Ontology; BP, biological process; CC, cellular component; MF, molecular function; PPI, protein-protein interaction; DEGs, Differentially expressed genes; NL, Non-Lymphatic metastasis; L, Lymphatic metastasis; N, Normal.

In order to study mechanisms underlying the role of HSP90AA1 in LM of HNSCC, we analyzed the expression profile and clinical data of HSP90AA1 in 419 cases of HNSCC (including 270 cases with LM and 149 cases without LM) and 44 cases of normal tissues from the TCGA database. As shown in Fig. 5A. The expression of HSP90AA1 was significantly up-regulated in HNSCC samples compared with normal samples. Meanwhile, the expression of HSP90AA1 in HNSCC samples with LM was higher than that in HNSCC samples without LM. These differences were statistically significant. This result is consistent with the expression of HSP90AA1 in HPSCC tissue specimens we completed previously. We divided the 419 HNSCC cases into high-expression and low-expression groups according to the expression level of HSP90AA1. 331 DEGs were screened out, which may be associated with HSP90AA1.According to the volcano plot with a cutoff of |log2FC| > 1, *p* < 0.05, a total of 264 up-regulated genes and 67 down-regulated genes were identified (Fig. 5B). The heatmap displays the top 50 genes that exhibit the most statistically significant differences between the HSP90AA1 high and low HSP90AA1 expression (Fig. 5C). A PPI network of these DEGs was constructed using the STRING database, setting the minimum required interaction score to 0.7. This network, encompassing 72 nodes and 72 edges, was visualized using Cytoscape software. Further PPI visualization and analysis were facilitated by employing the CytoHubba plugin within the Cytoscape software. Fig. 5D illustrates HSP90AA1 and CDH1 (E-Cadherin) exhibit strong mutual connectivity.

To illuminate the functions and pathways enriched by HSP90AA1, GO enrichment analysis was conducted on these core genes. This analysis yielded a total of 238 significant GO terms, which included 171 Biological Processes (BP), 25 Cellular Components (CC), and 42 Molecular Functions (MF). The top 10 significant enrichment terms in BP, CC, and MF with the highest gene counts are depicted in Fig. 5E. With regards to BP, these core genes demonstrated significant enrichment in processes such as epidermal cell differentiation, proliferation, and cell-cell adhesion. As for MF, these genes were primarily localized to the cytoskeleton, extracellular matrix, and endocytic vesicles. Concerning CC, these genes were largely enriched in functions related to oxidoreductase activity and monooxygenase activity. As shown in circos diagram of MF (Fig. 5F), HSP90AA1 and CDH1 (E-Cadherin) were highly enriched in the cytoskeleton, suggesting that HSP90AA1 may profoundly change the characteristics of tumor cells through the cytoskeleton, thus causing the epithelial tumor cells may EMT. In further studies, we analyzed the expression profile data of the above 419 HNSCC samples. The expression of CDH1 was significantly different between the LM group and the non-metastasis group (Fig. 5G). The difference and correlation expression showed that CDH1, CDH2 (N-Cadherin), SNAI2 (Slug), and SNAI1 (Snail) were significantly correlated with HSP90AA1 expression. The expression of CDH1 (E-cadherin) was negatively correlated with HSP90AA1, and CDH2 (N-Cadherin), SNAI2 (Slug), and SNAI1 (Snail) were all positively correlated with HSP90AA1 (Fig. 5H).

### Down-regulation of HSP90AA1 inhibits proliferation, migration,and invasive behavior of FaDu cells, and promotes cell apoptosis

**Figure 6:**
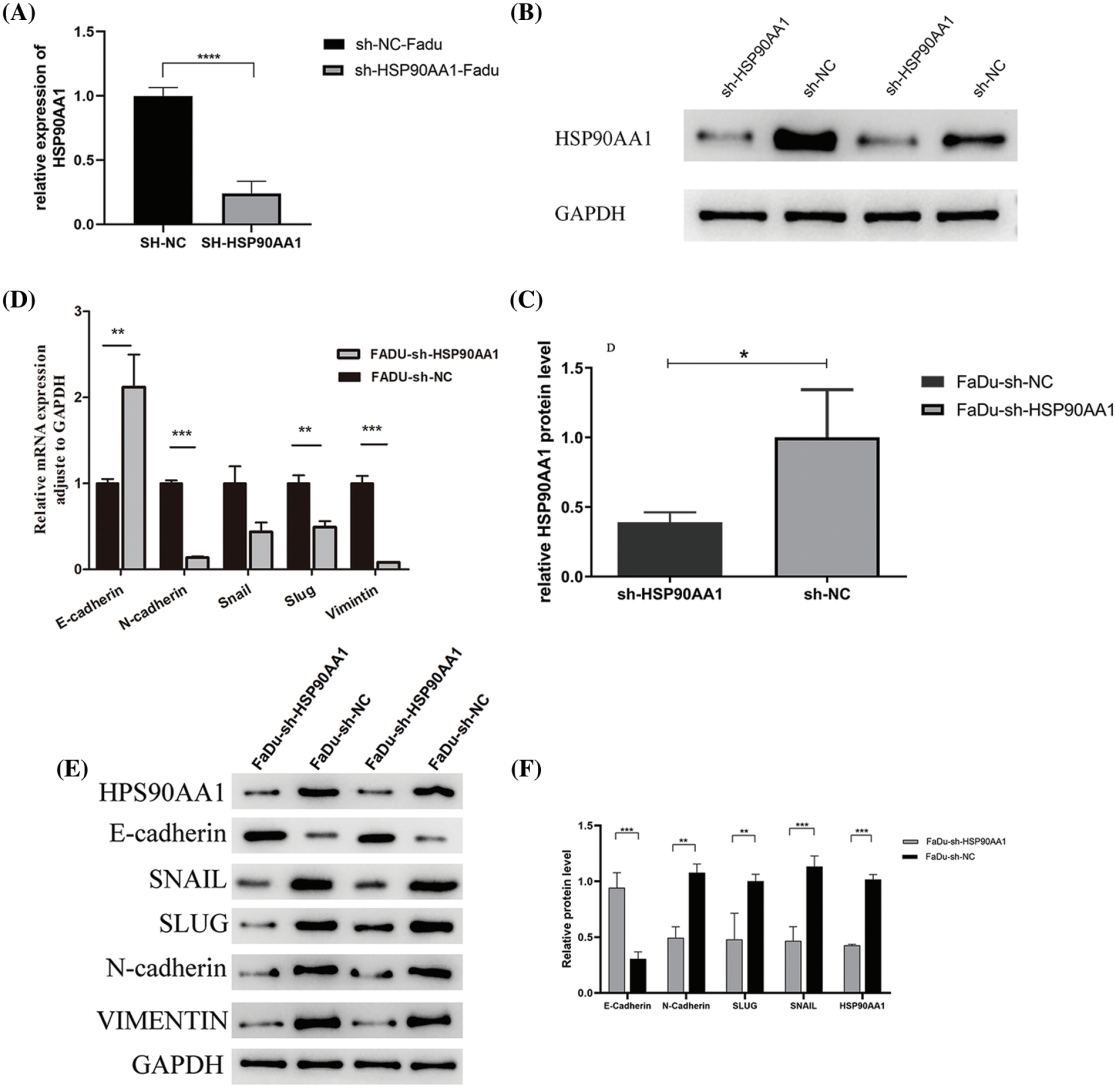
Expression of HSP90AA1 and EMT pathway markers in FaDu cell lines in the knockdown group (sh-HSP90AA1) and control group (sh-NC). (A) qRT-PCR assay of HSP90AA1, (B and C) Expression of HSP90AA1 protein level by western blot assay,protein expression levels were analyzed by Image J software. (D) qRT-PCR assay of E-cadherin, N-cadherin, SNAIL, SLUG, and VIMENTIN. (E and F) WB assay of HSP90AA1, E-cadherin, N-cadherin, SNAIL, SLUG, and VIMENTIN. Abbreviations: sh-RNA, short hairpin RNA; NC, negative control; FaDu-sh-NC, Lentivirus transfection reagent negative control Fadu cell line; FaDu-sh-HSP90AA1, Lentivirus transfected Fadu cell line with HSP90AA1 knockdown. **p < 0.01, ***p < 0.001, All the data are presented as mean ± SD from three independently performed experiments.

The suppression of HSP90AA1 was achieved *via* lentivirus shRNA transfection of FaDu cell lines, Evidenced by qRT-PCR and WB (Figs. 6A–6C), both mRNA and protein levels of HSP90AA1 were significantly decreased in the sh-HSP90AA1 group compared to the sh-NC group. Concurrently, with the inhibition of HSP90AA1 in FaDu cell lines, there was a surge in the mRNA expression of the EMT pathway marker E-cadherin, while the mRNA quantities of N-cadherin, SLUG, and VIMENTIN were reduced (Fig. 6D). Moreover, with the inhibition of HSP90AA1 in FaDu cell lines, the protein levels of E-cadherin upregulated, while the protein level of N-cadherin, SLUG, and SNAIL was reduced (Figs. 6E and 6F). These observations insinuate that HSP90AA1 could potentially enhance HPSCC LNMs through EMT suppression.

**Figure 7:**
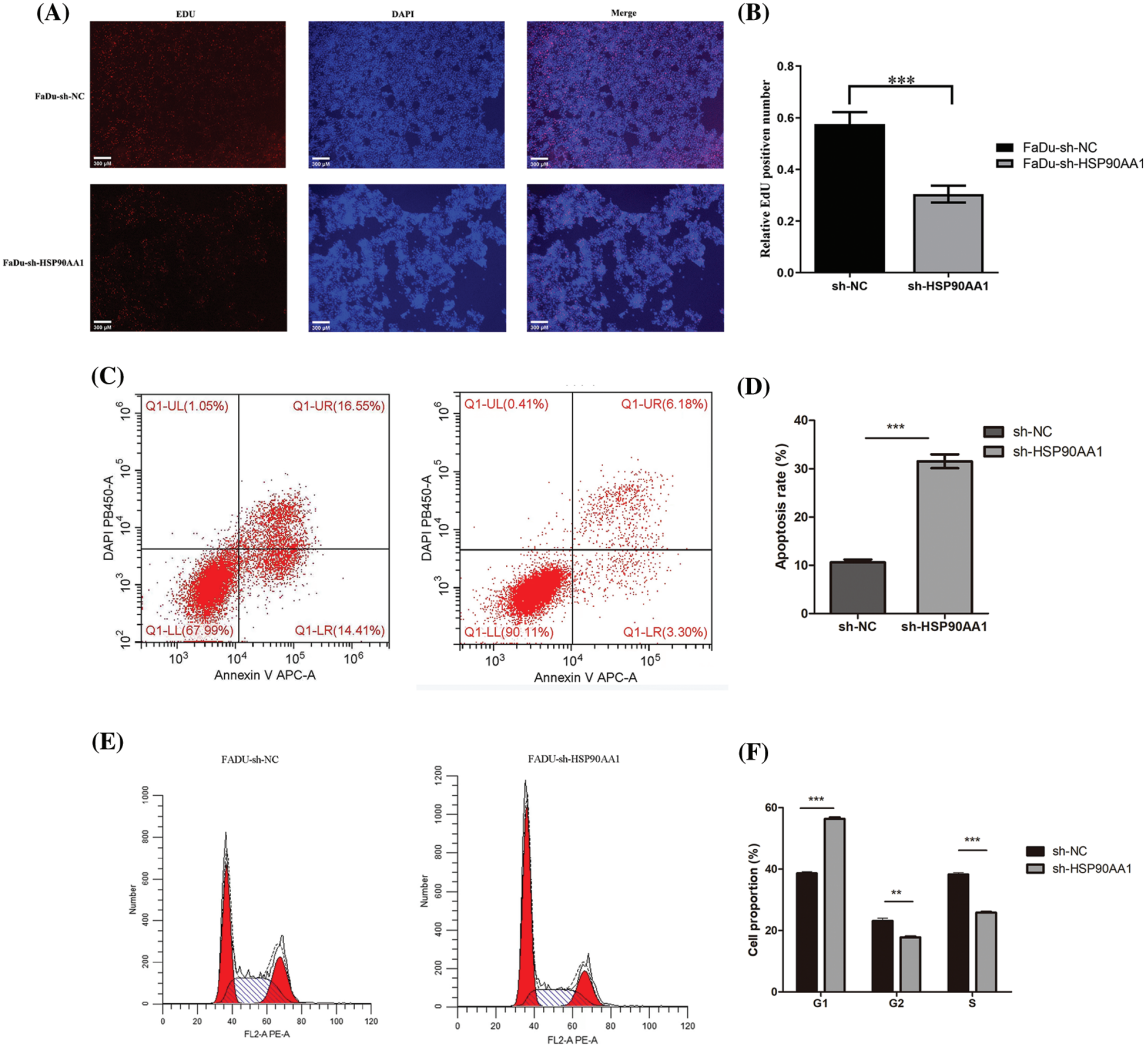
EDU and flow cytometry. (A) EdU proliferation assay in FaDu cell line. (B) Results analysis of EdU assay. (C) Apoptosis assay in FADU cell lines. (D) Results analysis of Apoptosis assay; (E) Cell cycle assay in FaDu cell lines. (F) Cell cycle assay analysis. Abbreviations: sh-RNA, short hairpin RNA; NC, negative control; FaDu-sh-NC, Lentivirus transfection reagent negative control Fadu cell line; FaDu-sh-HSP90AA1, Lentivirus transfected Fadu cell line with HSP90AA1 knockdown. **p < 0.01, ***p < 0.001, All the data are presented as mean ± SD from three independently performed experiments.

As depicted in Figs. 7A and 7B, the results of the EdU assay suggest that the down-regulation of HSP90AA1 expression can curtail the cell proliferation capacity Flow cytometry assays reveal a significant upsurge in the apoptosis rate in the HSP90AA1 knockdown group compared to the NC groups (Figs. 7C and 7D). Furthermore, in the HSP90AA1-suppressed group, there was a noticeable increase in G1 phase cells, while G2 and S phase cells exhibited a decrease. (Figs. 7E and 7F).

**Figure 8:**
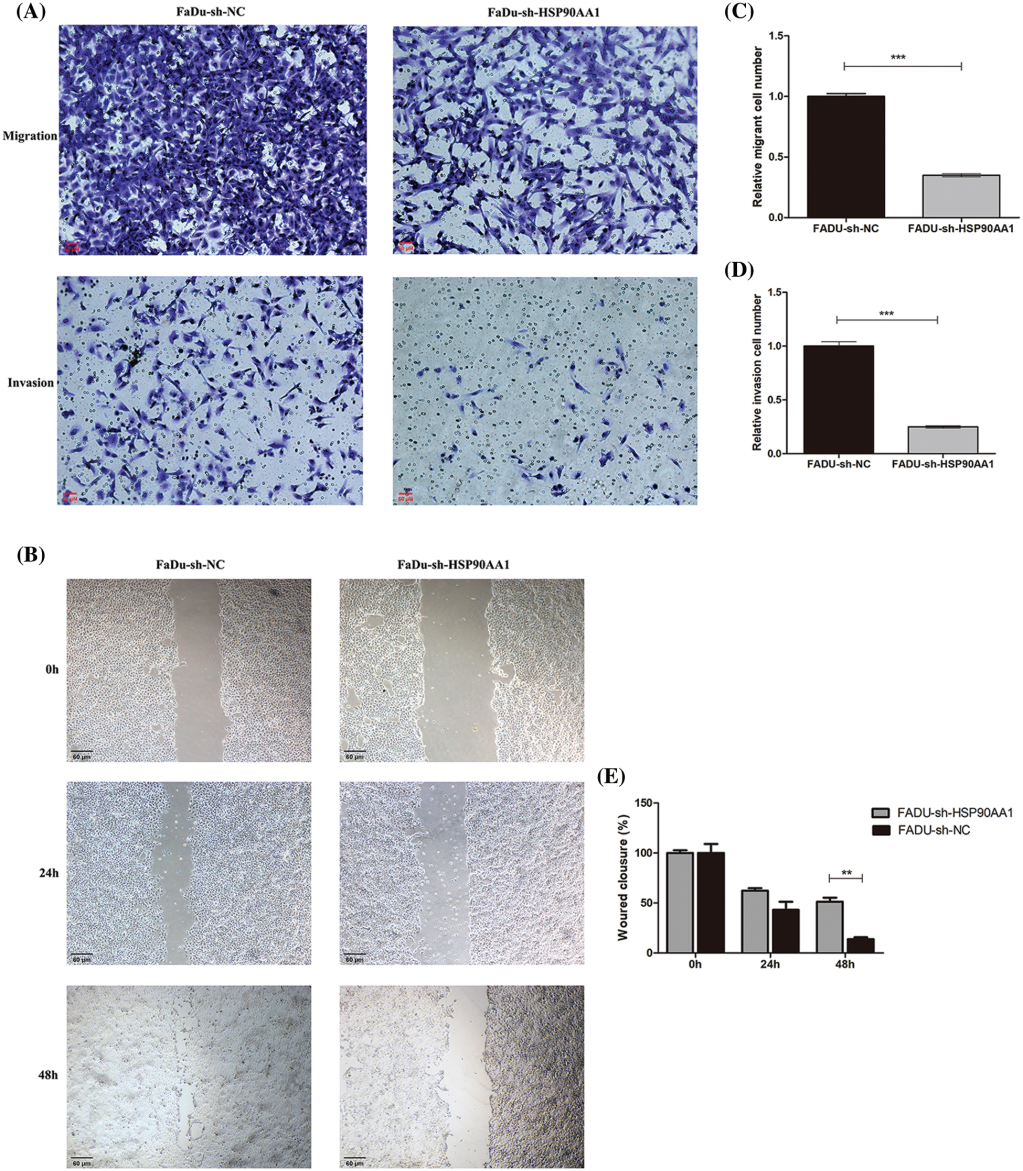
Transwell assay and Wound Healing assay. (A) Cell migration and invasion assay in FaDu cell line. (B) Wound Healing assay in FaDu cell line; (C, D) Results analysis of Transwell assay. (E) Results analysis of Wound Healing assay. Abbreviations: sh-RNA, short hairpin RNA; NC, negative control; FaDu-sh-NC, Lentivirus transfection reagent negative control Fadu cell line; FaDu-sh-HSP90AA1, Lentivirus transfected Fadu cell line with HSP90AA1 knockdown. **p < 0.01, ***p < 0.001. All the data are presented as mean ± SD from three independently performed experiments.

The outcomes of Transwell and Wound Healing assays indicated a significant reduction in cell migration and invasion abilities following the HSP90AA1 knockdown (Fig. 8). In summary, diminishing the expression of HPS90AA1 can significantly curb the proliferation, migration, and invasion of tumor cells while enhancing the apoptosis of FaDu cells.

#### Down-regulation of HSP90AA1 can inhibit tumor growth and LM

**Figure 9:**
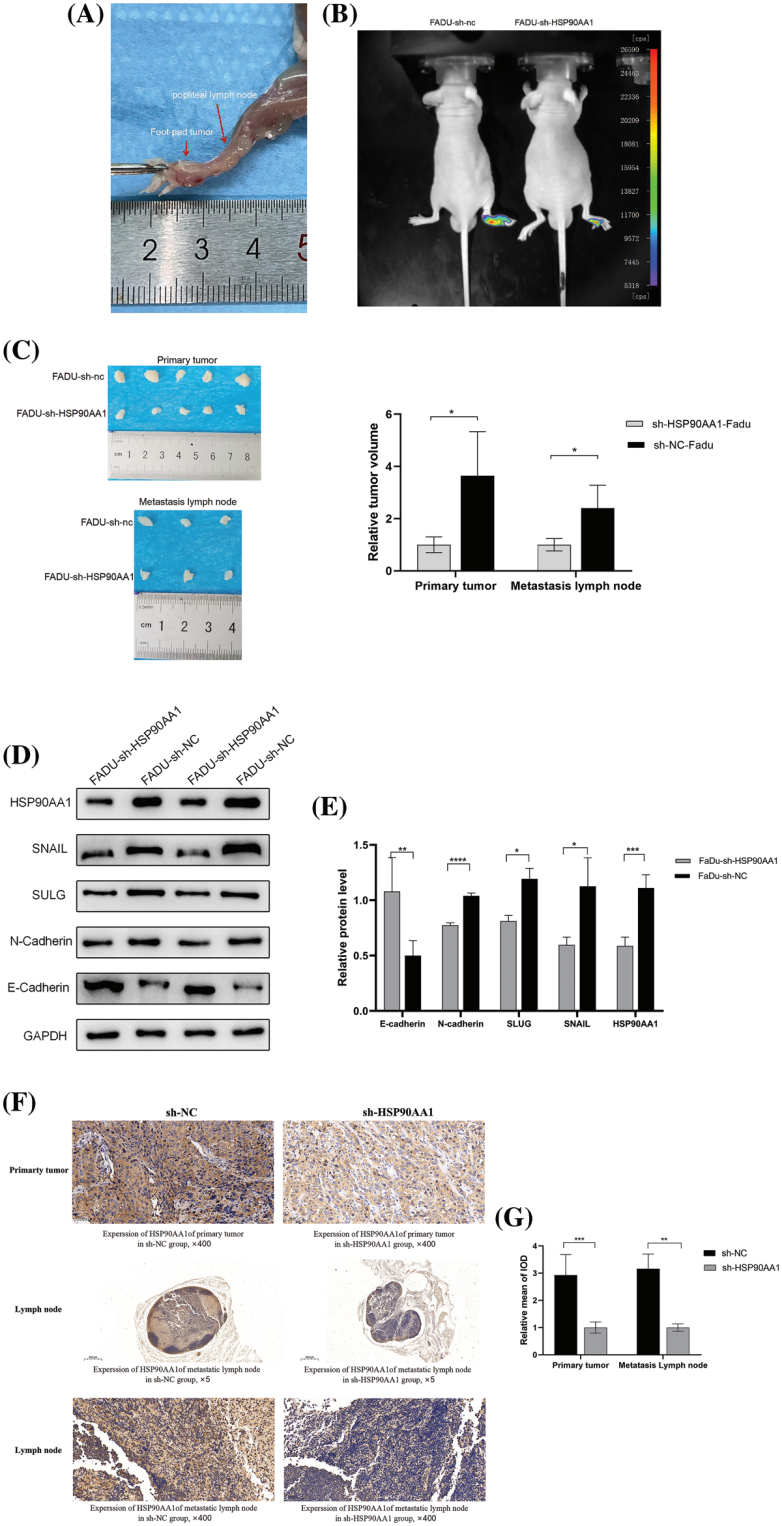
HSP90AA1 knockdown suppresses LM by regulating EMT *in vivo*. (A) Representative images of the nude mouse foot xenotransplantation model with lymph node metastasis. (B) Fluorescence imaging of nude mice. (C) Anatomy of primary footpad tumors and metastatic lymph nodes in nude mice, the right panel shows the statistical analysis of tumor volume. (D and E) WB assay of HPS90AA1, E-Cadherin, N-cadherin, SLUG, and SNAIL; GAPDH functioned as the internal control. (G) The mean staining intensity of primary tumor and metastatic lymph node. (F) Immunohistochemical staining for HSP90AA1in nude mice’s primary tumor and metastatic lymph node. Abbreviations: sh-RNA, short hairpin RNA; NC, negative control; FaDu-sh-NC, Nude mice injected with lentivirus transfection reagent negative control Fadu cell line; FaDu-sh-HSP90AA1, Nude mice injected with lentivirus transfected Fadu cell line with HSP90AA1 knockdown. *p < 0.05, **p < 0.01, ***p < 0.001, ****p < 0.0001. All the data are presented in the form of mean ± SD from three independently performed experiments.

The role of HSP90AA1 in LM of HPSCC was further clarified by creating a xenotransplantation model using the foot of a nude mouse (Fig. 9A). Two types of stably infected FaDu cells were injected into the mouse footpad, resulting in two groups: FaDu-sh-NC, and FaDu-sh-HSP90AA1 with each group comprising ten mice. After 28 days in SPF conditions, fluorescence imaging was conducted. Visually, it was evident that the expression of HSP90AA1 significantly curtailed both growth and LM of FaDu cells (Fig. 9B).

Upon anesthesia and euthanization of the mice, primary footpad tumors of foot-pad and MLNs were collected. Concurrently, tumor volumes and weights were measured and the number of MLNs was tallied per group (Fig. 9C). Notably, both footpad tumors and popliteal lymph nodes(LNs) were lighter and smaller in the HSP90AA1-suppressed group compared to the control group, indicating the inhibitory effect of HSP90AA1 on HPSCC tumorigenesis and LM. Subsequent validation of HSP90AA1 expression in primary tumors and LNs was carried out. WB assay (Figs. 9D and 9E) revealed significantly diminished HSP90AA1 protein levels in the HSP90AA1-suppressed group relative to the control group. Moreover, protein levels of EMT markers, such as N-cadherin, SLUG, and SNAIL mirrored the expression trend of HSP90AA1, while E-cadherin levels showed an inverse relationship. IHC findings further suggested a lower HSP90AA1 expression in the HSP90AA1-suppressed group than in the control group, both in primary tumors and MLNs (Figs. 9F and 9G, and Table 5). These observations imply that dampening HSP90AA1 expression can hinder tumor growth and LM.

**Table 5:** The immunohistochemical expression of HSP90AA1 in specimens of nude mice

Tissue	Number of nude mice	Expression of HSP90AA1	
		Positive	Negative
Primary tumor in the sh-NC group	10	8	2
Primary tumor in the sh-HSP90AA1 group	10	4	6
Metastatic lymph node in sh-NC group	3	3	0
Metastatic lymph node in sh-HSP90AA1 roup	3	1	2

## Discussion

The hypopharyngeal region, with its extensive anastomosis between parallel lymphatic systems and abundant lymphatic drainage, provides a significant anatomical basis for LNMs in HPSCC. This complex lymphatic network increases the likelihood of LNMs in HPSCC patients [35,36]. Previous studies have illustrated that LNMs—particularly factors such as diameter, quantity, and invasion of MLNs are independent prognostic risk factors for HPSCC [9,10,37]. Similar conclusions were derived from our study, which employed KMSA, LR, and CRA on patients’ clinicopathological characteristics. Therefore, researching the mechanism behind LNMs in HPSCC and identifying associated biomarkers bears substantial theoretical and practical importance. These insights could potentially enhance the precision of HPSCC treatment and improve the survival rate of HPSCC patients.

In our previous studies, transcriptome sequencing of clinical specimens has demonstrated that HSP90AA1 is a significantly differential gene in HPSCC patients with LNMs. Indeed, the upregulation of HSP90AA1 was associated with the occurrence, invasion, metastasis, and progression of various malignant tumors [38,39]. At the same time, it has also been reported that HSP90AA1 may promote malignant tumor LM by regulating EMT [33,34]. In this study, the significantly up-regulated HSP90AA1 was closely related to some clinicopathological characteristics in HPSCC patients with LNMs, such as extracapsular extension and N staging. Additionally, patients with high HSP90AA1 expression had a shorter OS. ROC curves showed that the expression of HSP90AA1 could be a reliable indicator to predict the metastasis risk and prognosis of HPSCC. In this study, by analyzing the expression profile and clinical data of HNSCC in the TCGA database, we found that HSP90AA1 may profoundly change the characteristics and of polarity tumor cells through cytoskeleton,thus decreasing adhesion among cell-to-cell and increasing the invasiveness of tumor cells. In FaDu cell lines transfected with lentivirus knockout HSP90AA1, the expression of E-cadherin increased and N-cadherin, SNAIL, SLUG, and VIMENTIN expression decreased. Meanwhile, the proliferation, migration, and invasion, promoting cell apoptosis of tumor cells were significantly inhibited and suggested that HSP90AA1 may inhibit the metastasis of tumor cells by regulating EMT. The results of *in vivo* experiments also indicate that HSP90AA1 is closely related to EMT and plays a crucial role in LNMs of HPSCC.

In this study, we screened out the differential gene HSP90AA1 that affected the LM of HPSCC through gene sequencing, and further explored its expression and prognostic value in HPSCC tissues, suggesting that HSP90AA1 might be a biomarker for predicting the metastasis biomarker and unique treatment target of HPSCC. Additionally, we preliminarily investigated the mechanism of HSP90AA1 in the progression and LNMs of HPSCC, and found that HSP90AA1 might promote LNMs of HPSCC by regulating EMT. Hence, future studies shall achieve the prediction of LNMs and prognosis of HPSCC with HSP90AA1 as the tumor biomarker. Additionally, HSP90AA1 might regulate EMT thus leading to LM, which provides a potential target for HPSCC treatment.

## Data Availability

All data generated or analyzed during this study are included in this published article.

## Abbreviations

LM: Lymphatic metastasis
HPSCC: Hypopharyngeal squamous cell carcinoma
WB: Western-blotting
IHC: Immunohistochemistry
KMSA: Kaplan–Meier survival analysis
LR: Log-rank test
CRA: Cox’s regression analysis
HNSCC: Head and neck squamous cell carcinoma
EMT: Epithelial-mesenchymal transition
LNMs: Lymph node metastasis
5YSR: Five-year survival rate
MLNs: Metastatic lymph nodes
SR: Survival rate
HSP90α: Shock protein 90 α
FAH-CQMU: The First Affiliated Hospital of Chongqing Medical University
PVDF: Polyvinylidene fluoride
NC: Negative control
qRT-PCR: Quantitative reverse transcription-polymerase chain reaction
FBS: Fetal bovine serum
sh-RNA: Short hairpin RNA
TNM: Tumor node metastasis
IOD: Integral optical density
OS: Overall survival
TCGA: The Cancer Genome Atlas
PPI: Protein-protein interaction
DEGs: Differentially expressed genes
ROC: Receiver operating characteristic
BP: Biological processes
CC: Cellular component
MF: Molecular function
MOI: Multiplicity of Infection
SPF: Specific pathogen-free
FC: Fold change
GO: Gene Ontology

## References

[1] Forastiere, A. A., Goepfert, H., Maor, M., Pajak, T. F., Weber, R. et al. (2003). Concurrent chemotherapy and radiotherapy for organ preservation in advanced laryngeal cancer. The New England Journal of Medicine*,* 349 *(*22*),* 2091–2098. 10.1056/NEJMoa031317; 14645636

[2] Kiyosawa, N., Manabe, S., Yamoto, T., Sanbuissho, A. (2010). Practical application of toxicogenomics for profiling toxicant-induced biological perturbations. International Journal of Molecular Sciences*,* 11 *(*9*),* 3397–3412. 10.3390/ijms11093397; 20957103 PMC2956103

[3] Ferris, R. L., Licitra, L. (2019). PD-1 immunotherapy for recurrent or metastatic HNSCC. The Lancet*,* 394 *(*10212*),* 1882–1884. 10.1016/S0140-6736(19)32539-5; 31679948

[4] Saada-Bouzid, E., Peyrade, F., Guigay, J. (2019). Immunotherapy in recurrent and or metastatic squamous cell carcinoma of the head and neck. Current Opinion in Oncology*,* 31 *(*3*),* 146–151. 10.1097/CCO.0000000000000522; 30893146

[5] Ahn, D., Kim, J. H., Sohn, J. H., Sin, C. M., Lee, J. E. (2013). Laryngeal preservation in stage III/IV resectable laryngo-hypopharyngeal squamous cell carcinoma following concurrent chemoradiotherapy with capecitabine/cisplatin. Molecular and Clinical Oncology*,* 1 *(*4*),* 685–691. 10.3892/mco.2013.113; 24649229 PMC3915701

[6] Li, M., Lorenz, R. R., Khan, M. J., Burkey, B. B., Adelstein, D. J. et al. (2013). Salvage laryngectomy in patients with recurrent laryngeal cancer in the setting of nonoperative treatment failure. Otolaryngology-Head and Neck Surgery*,* 149 *(*2*),* 245–251. 10.1177/0194599813486257; 23585149

[7] Aupérin, A. (2020). Epidemiology of head and neck cancers: An update. Current Opinion in Oncology*,* 32 *(*3*),* 178–186. 10.1097/CCO.0000000000000629; 32209823

[8] Siegel, R. L., Miller, K. D., Fuchs, H. E., Jemal, A. (2021). Cancer statistics, 2021. CA: A Cancer Journal for Clinicians*,* 71 *(*1*),* 7–33; 33433946 10.3322/caac.21654

[9] Spector, J. G., Sessions, D. G., Haughey, B. H., Chao, K. S., Simpson, J. et al. (2001). Delayed regional metastases, distant metastases, and second primary malignancies in squamous cell carcinomas of the larynx and hypopharynx. Laryngoscope*,* 111 *(*6*),* 1079–1087. 10.1097/00005537-200106000-00028; 11404625

[10] Roberts, T. J., Colevas, A. D., Hara, W., Holsinger, F. C., Oakley-Girvan, I. et al. (2016). Number of positive nodes is superior to the lymph node ratio and American Joint Committee on Cancer N staging for the prognosis of surgically treated head and neck squamous cell carcinomas. Cancer*,* 122 *(*9*),* 1388–1397. 10.1002/cncr.29932; 26969807

[11] Xing, Y., Zhang, J., Lin, H., Gold, K. A., Sturgis, E. M. et al. (2016). Relation between the level of lymph node metastasis and survival in locally advanced head and neck squamous cell carcinoma. Cancer*,* 122 *(*4*),* 534–545. 10.1002/cncr.29780; 26554754 PMC4742373

[12] Barroso Ribeiro, R., Ribeiro Breda, E., Fernandes Monteiro, E. (2012). Prognostic significance of nodal metastasis in advanced tumours of the larynx and hypopharynx. Acta Otorrinolaringologica (English Edition)*,* 63 *(*4*),* 292–298. 10.1016/j.otoeng.2012.07.014 22579383

[13] Kuo, P., Mehra, S., Sosa, J. A., Roman, S. A., Husain, Z. A. et al. (2016). Proposing prognostic thresholds for lymph node yield in clinically lymph node-negative and lymph node-positive cancers of the oral cavity. Cancer*,* 122 *(*23*),* 3624–3631. 10.1002/cncr.v122.23 27479645

[14] Antonio, J. K., Santini, S., Politi, D., Sulfaro, S., Spaziante, R. et al. (2012). Sentinel lymph node biopsy in squamous cell carcinoma of the head and neck: 10 years of experience. Acta Otorhinolaryngologica Italica*,* 32 *(*1*),* 18–25; 22500062 PMC3324960

[15] Grasl, S., Janik, S., Parzefall, T., Formanek, M., Grasl, M. C. et al. (2020). Lymph node ratio as a prognostic marker in advanced laryngeal and hypopharyngeal carcinoma after primary total laryngopharyngectomy. Clinical Otolaryngology*,* 45 *(*1*),* 73–82. 10.1111/coa.13468; 31660699

[16] Kalluri, R., Neilson, E. G. (2003). Epithelial-mesenchymal transition and its implications for fibrosis. Journal of Clinical Investigation*,* 112 *(*12*),* 1776–1784. 10.1172/JCI200320530 14679171 PMC297008

[17] Kalluri, R., Weinberg, R. A. (2009). The basics of epithelial-mesenchymal transition. Journal of Investigation*,* 119 *(*6*),* 1420–1428. 10.1172/JCI39104; 19487818 PMC2689101

[18] Valastyan, S., Weinberg, R. A. (2011). Tumor metastasis: Molecular insights and evolving paradigms. Cell*,* 147 *(*2*),* 275–292. 10.1016/j.cell.2011.09.024; 22000009 PMC3261217

[19] Maeda, M., Johnson, K. R., Wheelock, M. J. (2005). Cadherin switching: Essential for behavioral but not morphological changes during an epithelium-to-mesenchyme transition. Journal of Cell Science*,* 118, 873–887. 10.1242/jcs.01634; 15713751

[20] Vogelstein, B., Fearon, E. R., Hamilton, S. R., Kern, S. E., Preisinger, A. C. et al. (1998). Genetic alterations during colorectal-tumor development. New England Journal of Medicine*,* 319 *(*9*),* 525–532. 10.1056/NEJM198809013190901; 2841597

[21] D’Uva, G., Bertoni, S., Lauriola, M., de Carolis, S., Pacilli, A. et al. (2013). Beta-catenin/HuR post-transcriptional machinery governs cancer stem cell features in response to hypoxia. PLoS One*,* 8 *(*11*),* e80742. 10.1371/journal.pone.0080742; 24260469 PMC3829939

[22] Jung, C. H., Kim, J., Park, J. K., Hwang, S. G., Moon, S. K. et al. (2013). Mdm2 increases cellular invasiveness by binding to and stabilizing the Slug mRNA. Cancer Letters*,* 335 *(*2*),* 270–277. 10.1016/j.canlet.2013.02.035; 23438693

[23] Recouvreux, M. V., Moldenhauer, M. R., Galenkamp, K. M. O., Jung, M., James, B. et al. (2020). Glutamine depletion regulates Slug to promote EMT and metastasis in pancreatic cancer. Journal of Experimental Medicine*,* 217 *(*9*),* e20200388. 10.1084/jem.20200388; 32510550 PMC7478719

[24] Menyhárt, O., Győrffy, B. (2021). Multi-omics approaches in cancer research with applications in tumor subtyping, prognosis, and diagnosis. Computational and Structural Biotechnology Journal*,* 19 *(*11*),* 949–960. 10.1016/j.csbj.2021.01.009; 33613862 PMC7868685

[25] Li, Y., Lu, T., Hu, G. (2020). Gene sequencing and expression of Raf-1 in lymphatic metastasis of hypopharyngeal carcinoma. Cancer Biomarkers*,* 28 *(*2*),* 181–191. 10.3233/CBM-191238; 32224526 PMC12662343

[26] Li, Y., Pan, M., Lu, T., Yu, D., Liu, C. et al. (2022). RAF1 promotes lymphatic metastasis of hypopharyngeal carcinoma via regulating LAGE1: An experimental research. Journal of Translational Medicine*,* 20 *(*1*),* 255. 10.1186/s12967-022-03468-7; 35668458 PMC9172115

[27] Chatterjee, S., Burns, T. F. (2017). Targeting heat shock proteins in cancer: A promising therapeutic approach. International Journal of Molecular Sciences*,* 18 *(*9*),* 1978. 10.3390/ijms18091978; 28914774 PMC5618627

[28] Welch, W. J., Feramisco, J. R. (1982). Purification of the major mammalian heat shock proteins. Journal of Biological Chemistry*,* 257 *(*24*),* 14949–14959. 10.1016/S0021-9258(18)33376-3 7174676

[29] Miyata, Y., Nakamoto, H., Neckers, L. (2013). The therapeutic target Hsp90 and cancer hallmarks. Current Pharmaceutical Design*,* 19 *(*3*),* 347–365. 10.2174/138161213804143725; 22920906 PMC7553218

[30] Li, Y., Zhang, T., Schwartz, S. J., Sun, D. (2009). New developments in Hsp90 inhibitors as anti-cancer therapeutics: Mechanisms, clinical perspective and more potential. Drug Resistance Updates*,* 12 *(*1–2*),* 17–27. 10.1016/j.drup.2008.12.002; 19179103 PMC2692088

[31] Banerji, U. (2009). Heat shock protein 90 as a drug target: Some like it hot. Clinical Cancer Research*,* 15 *(*1*),* 9–14. 10.1158/1078-0432.CCR-08-0132; 19118027

[32] Lee, H. W., Kim, K. M. (2019). Clinical significance of heat shock protein 90α expression as a biomarker of prognosis in patients with gastric cancer. Nigerian Journal of Clinical Practice*,* 22 *(*12*),* 1698–1705. 10.4103/njcp.njcp_68_19; 31793477

[33] Chong, K. Y., Kang, M., Garofalo, F., Ueno, D., Liang, H. et al. (2019). Inhibition of heat shock protein 90 suppresses TWIST1 transcription. Molecular Pharmacology*,* 96 *(*2*),* 168–179. 10.1124/mol.119.116137; 31175180

[34] Nagaraju, G. P., Long, T. E., Park, W., Landry, J. C., Taliaferro-Smith, L. et al. (2015). Heat shock protein 90 promotes epithelial to mesenchymal transition, invasion, and migration in colorectal cancer. Molecular Carcinogenesis*,* 54 *(*10*),* 1147–1158. 10.1002/mc.22185; 24861206

[35] Habib, A. (2018). Management of advanced hypopharyngeal carcinoma: Systematic review of survival following surgical and non-surgical treatments. Journal of Laryngology and Otology*,* 132 *(*5*),* 385–400. 10.1017/S0022215118000555; 29891019

[36] Schmukler, E., Grinboim, E., Schokoroy, S., Amir, A., Wolfson, E. et al. (2013). Ras inhibition enhances autophagy, which partially protects cells from death. Oncotarget*,* 4 *(*1*),* 145–155. 10.18632/oncotarget.703; 23370967 PMC3702214

[37] Zhan, F., Barlogie, B., Arzoumanian, V., Huang, Y., Williams, D. R. et al. (2007). Gene-expression signature of benign monoclonal gammopathy evident in multiple myeloma is linked to good prognosis. Blood*,* 109 *(*4*),* 1692–1700. 10.1182/blood-2006-07-037077; 17023574 PMC1794073

[38] Closa, A., Cordero, D., Sanz-Pamplona, R., Solé, X., Crous-Bou, M. et al. (2014). Identification of candidate susceptibility genes for colorectal cancer through eQTL analysis. Carcinogenesis*,* 35 *(*9*),* 2039–2046. 10.1093/carcin/bgu092; 24760461 PMC4146415

[39] Mangangcha, I. R., Malik, M. Z., Küçük, Ö., Ali, S., Singh, R. K. B. (2019). Identification of key regulators in prostate cancer from gene expression datasets of patients. Scientific Reports*,* 9 *(*1*),* 16420. 10.1038/s41598-019-52896-x; 31712650 PMC6848149

